# Analyses of long non-coding RNA and mRNA profiling in the spleen of diarrheic piglets caused by *Clostridium perfringens* type C

**DOI:** 10.7717/peerj.5997

**Published:** 2018-11-30

**Authors:** Zunqiang Yan, Xiaoyu Huang, Wenyang Sun, Qiaoli Yang, Hairen Shi, Tiantuan Jiang, Shenggui Li, Pengfei Wang, Shuangbao Gun

**Affiliations:** 1 College of Animal Science and Technology, Gansu Agricultural University, Lanzhou, Gansu, China; 2 Gansu Research Center for Swine Production Engineering and Technology, Lanzhou, Gansu, China

**Keywords:** Swine, Diarrhea, LncRNA, *Clostridium perfringens* type C, Spleen

## Abstract

**Background:**

*Clostridium perfringens* (*C. perfringens*) type C is the most common bacteria causing piglet diarrheal disease and it greatly affects the economy of the global pig industry. The spleen is an important immune organ in mammals; it plays an irreplaceable role in resisting and eradicating pathogenic microorganisms. Based on different immune capacity in piglets, individuals display the resistance and susceptibility to diarrhea caused by *C. perfringens* type C. Recently, long non-coding RNA (lncRNA) and mRNA have been found to be involved in host immune and inflammatory responses to pathogenic infections. However, little is known about spleen transcriptome information in piglet diarrhea caused by *C. perfringens* type C.

**Methods:**

Hence, we infected 7-day-old piglets with *C. perfringens* type C to lead to diarrhea. Then, we investigated lncRNA and mRNA expression profiles in spleens of piglets, including control (SC), susceptible (SS), and resistant (SR) groups.

**Results:**

As a result, 2,056 novel lncRNAs and 2,417 differentially expressed genes were found. These lncRNAs shared the same characteristics of fewer exons and shorter length. Bioinformatics analysis identified that two lncRNAs (*ALDBSSCT0000006918* and *ALDBSSCT0000007366*) may be involved in five immune/inflammation-related pathways (such as Toll-like receptor signaling pathway, MAPK signaling pathway, and Jak-STAT signaling pathway), which were associated with resistance and susceptibility to *C. perfringens* type C infection. This study contributes to the understanding of potential mechanisms involved in the immune response of piglets infected with *C. perfringens* type C.

## Introduction

*Clostridium perfringens* (*C. perfringens*) is a spore-forming Gram-positive, anaerobic bacillus that can be divided into five types (A to E) based on the production of four main toxins (α, β, ε, ι) (Petit, Gibert & Popoff, 1999). It causes numerous gastrointestinal infections, gas gangrene, and food poisoning with high morbidity in both humans and animals (Bryant, 2003; Brynestad & Granum, 2002; Songer & Uzal, 2005). Recently, this pathogen has also been recorded as the leading cause of foodborne infection outbreaks in many countries, including the USA (Scallan et al., 2011), England (Gormley et al., 2011), and Japan (Komatsu et al., 2012). *C. perfringens* type C causes hemorrhagic inflammation, necrotic enteritis, and death in piglets (Uzal & McClane, 2011); it is a leading cause of piglet diarrheal disease, and results in significant economic loss to the global pig industry (Schaefer et al., 2012; Schaefer, Zimmermann & Posthaus, 2013; Wollschlager et al., 2009). *C. perfringens* type C infections are acquired through the mouth and can produce lethal α and β toxins in the intestines of piglets (Songer & Uzal, 2005). As the disease progresses, the toxins are absorbed in the small intestine and are transferred to the spleen and other organs causing systemic damage through blood and lymph circulation (Songer & Uzal, 2005). In addition, consumption of poorly prepared meat contaminated with this bacterium can result in human food poisoning (Brynestad & Granum, 2002; Lawrence & Walker, 1976). Therefore, there is an urgent need to clarify the molecular mechanisms underlying pathogenesis in hosts, and to ultimately offer better strategies of disease control.

Non-protein-coding transcripts of more than 200 nucleotides in length are called long non-coding RNAs (lncRNAs). Studies have found that some lncRNAs are involved in host immune and inflammatory responses to pathogen infections or their products. In lipopolysaccharide-treated THP-1 cells (human monocyte), lncRNA *IL7R* (human interleukin-7 receptor) can reduce the expression of proinflammatory mediators (such as *IL-8* and *IL-6*) to alleviate the inflammatory response (Cui et al., 2014). In *Mycobacterium tuberculosis* infected CD8^+^ T cells, lncRNA *CD244* (cluster of differentiation 244) is up-regulated by CD244 maintaining a permissive chromatin state. Up-regulated lncRNA *CD244* can recruit EZH2 (enhancer of zeste homolog 2) to mediate modification of a more repressive chromatin state at INF-γ and TNF-α loci, which inhibits *TNF-α* and *INF-γ* expression for *M. tuberculosis* proliferation (Wang et al., 2015). The down-regulated lncRNA *MEG3* (maternally expressed 3) acts as a functional regulator to eliminate *Mycobacterium bovis* Bacillus Calmette-Guerin (*M. bovis* BCG) via inducting autophagy in macrophages (Pawar et al., 2016). LincRNA *EPS* (erythroid prosurvival) can regulate immune related genes (such as *IL15*, *IL1α*, and *CXCL2*) to restrain inflammation in *Listeria monocytogenes* infected macrophages and dendritic cells (Atianand et al., 2016).

The spleen synthesizes antibodies to protect the body by modulating innate and adaptive immunity through filtering and responding to pathogens (Bronte & Pittet, 2013). Research has been conducted on the spleen following infection with different pathogens: splenomegaly is a characteristic of malaria caused by *Plasmodium* (Engwerda, Beattie & Amante, 2005); and spleen injury (apoptotic and proliferative alterations) may be induced by *Salmonella* (Li et al., 2017). Furthermore, studies have shown that the spleen plays a key role in *C. perfringens* type A-infection of poultry. In the spleen of broilers infected with *C. perfringens* type A, results reveal that some immune response pathways (such as major histocompatibility complex (MHC) class I and II and apoptosis pathways) are targeted (Zhou et al., 2009). It is known that *C. perfringens* type A produces α toxin and *C. perfringens* type C generates α and β toxins. Different types of *C. perfringens* may have different pathogeneses because of their various toxins and different hosts. Little is known about the biological function and significance of lncRNAs and mRNAs in the spleens of *C. perfringens* type C-infected piglets.

In this study, we analyzed transcriptome profiles of the spleen of diarrheic piglets (caused by *C. perfringens* type C). Our results enrich the transcript catalog (both mRNAs and lncRNAs) in porcine species, allowing us to screen potential mRNAs, lncRNAs, and pathways that are involved in the regulation of responses in piglets infected by *C. perfringens* type C. Our study may also provide a valuable resource for studying the lncRNAs that are involved in host defense against *C. perfringens* infection.

## Materials and Methods

### Ethics statement

All studies involving piglets were carried out in accordance with the regulations for the Administration of Affairs Concerning Experimental Animals (Ministry of Science and Technology, China; revised in June 2004). Sample collection was approved by the ethics committee of Gansu Agricultural University. Animals were humanely sacrificed to alleviate suffering.

### *C. perfringens* type C culture and animal treatment

A total of 30 healthy 7-day-old piglets (Y × L), that tested negative for *C. perfringens* type C, *Escherichia coli*, and *Salmonella*, were selected as experimental subjects. Of these, 25 piglets were randomly selected as the experimental group. *C. perfringens* type C strain (CVCC 2032) was purchased from the China Veterinary Culture Collection Center. Each piglet was dosed with one mL of bouillon culture-medium (HopeBio, Qingdao, China) inoculated with *C. perfringens* type C at 37 °C for 16 h, which approximated to 1 × 10^9^ Colony-Forming Unit (CFU) per mL; and they were inoculated by oral gavage once a day for 5 days. The remaining five piglets were given an equal volume of aseptic medium as the control group (SC). During the experiment, diarrheic times and fecal traits were recorded. Fecal symptom traits (0 = normal, solid feces; 1 = slight diarrhea, soft and loose feces; 2 = moderate diarrhea, semi-liquid feces; 3 = severe diarrhea, liquid and unformed feces) were judged and scored by a previously described method (Kelly, O’Brien & McCracken, 1990; Yang et al., 2013). A total of 25 piglets were ranked from high to low based on total diarrhea scores. The top five and bottom five piglets based on total diarrhea scores were considered as the susceptible group (SS) and resistant group (SR), respectively. Piglets in SS had more serious diarrhea than those in SR.

### Sample collection

Spleen and other tissues from 15 piglets were collected, flushed with phosphate buffer saline (PBS), frozen in liquid nitrogen, and stored at −80 °C until used for RNA isolation.

### Total RNA extraction and qualification

Total RNA was extracted using TRIzol reagent (Invitrogen, Carlsbad, CA, USA). Subsequently, spleen total RNA purity and quantity were measured using a NanoPhotometer spectrophotometer (Implen, Westlake Village, CA, USA) and Qubit 2.0 Fluorometer (Life Technologies, Carlsbad, CA, USA). In addition, RNA integrity was judged using the RNA Nano6000 Assay Kit through the Bioanalyzer 2100 system (Agilent Technologies, Santa Clara, CA, USA).

### Library preparation for sequencing

Approximately three μg of total RNA from each piglet was used as input material for RNA library preparations. First, rRNA-free total RNA was obtained by ethanol precipitation after removal of the ribosomal RNA using an Epicentre Ribo-zero™ rRNA Removal Kit (Epicentre, Madison, WI, USA). Then, the rRNA-depleted RNA was used to construct cDNA libraries using a NEBNext® Ultra™ Directional RNA Library Prep Kit for Illumina (NEB, Ipswich, MA, USA). The synthesis of the first strand cDNA was conducted using random hexamer primers and M-MuLV reverse transcriptase. Subsequently, the second strand cDNA was synthesized by DNA polymerase I with RNase H added to the reaction buffer supplied with the enzymes. After adenylation of 3′ ends of DNA fragments, the second strand cDNA was ligated with NEBNext adaptor with a hairpin loop structure. The cDNA fragments, 150~200 bp, were isolated using the AMPure XP system (Beckman Coulter, Beverly, NJ, USA). Lastly, to enrich the cDNA libraries, PCR amplification was performed using Phusion High-Fidelity DNA polymerase, Universal PCR primers, and Index Primer (NEB, Ipswich, MA, USA). Products were purified with an AMPure XP system (Beckman Coulter, Brea, CA, USA), and the quality of the library was checked using the Agilent Bioanalyzer 2100 system (Agilent Technologies, Santa Clara, CA, USA). The libraries were sequenced on an Hiseq 4000 platform (Illumina, San Diego, CA, USA) to generate PE150 reads at the Beijing Novogene Bioinformatics Institute in China.

### Mapping and transcriptome assembly

Raw data in fastq format were handled through in-house perl scripts. Clean data were obtained by removing adapters, over 10% poly-Ns (unrecognized bases), and low-quality (>50% of bases with Phred scores < 5) reads from raw reads. Meanwhile, the Phred score (Q20, Q30) and GC (Guanine and Cytosine) content of the clean data were evaluated. The paired-end clean reads were mapped to the pig genome sequence assembly (*Sus scrofa* 10.2) with Tophat (Trapnell, Pachter & Salzberg, 2009). The pig reference genome and gene model annotation files were downloaded from the pig genome website. The mapped reads were assembled using Cufflinks (Trapnell et al., 2012) and Scripture (Guttman et al., 2010). HTSeq (Anders, Pyl & Huber, 2015) was applied to evaluate the proportion of reads aligning to pseudogene, rRNA, miRNA, snRNA, misc-RNA, and so on.

### Gene expression quantification

Gene expression levels were calculated by FPKM (Fragments Per Kilobase Million) evaluated using Cuffdiff (Trapnell et al., 2010), which offers statistical methods for screening differential expression in digital transcriptomes. The gene expression level was calculated by FPKM evaluation using gene expression data. Biological replicates, transcripts, or genes with corrected *P*-value < 0.05 were treated as differentially expressed.

### LncRNA identification

**Figure 1:**
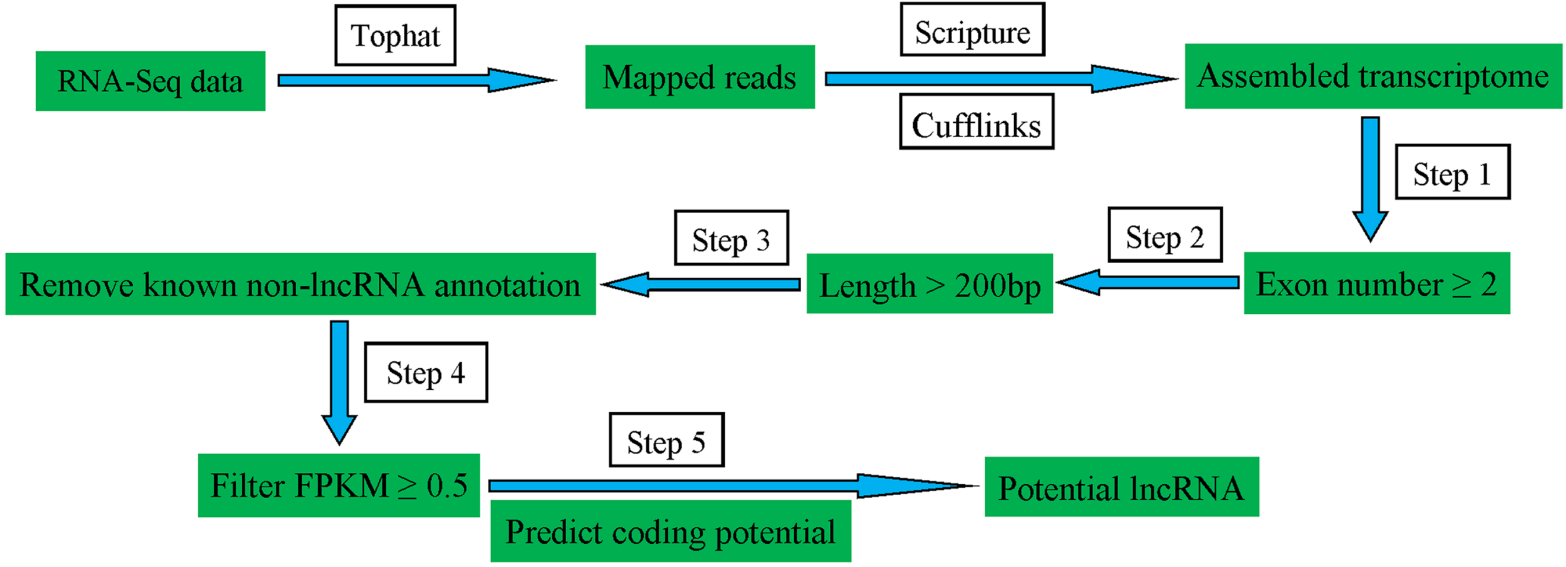
Schematic diagram of the pipeline for identifying lncRNAs.

To identify the lncRNAs expressed in pig spleens, a series of highly stringent filtering criteria was performed for discarding transcripts without the characteristics of lncRNAs (Fig. 1). Step 1: Low-read coverage and single exon transcripts were eliminated and exon number ≥ 2 transcripts were reserved; Step 2: Transcripts < 200 bp were removed; Step 3: Transcripts blasted to known mRNA, tRNA, rRNA, pseudogenes, miRNA, snRNA and snoRNA were excluded; Step 4: Transcripts of FPKM ≥ 0.5 were selected; Step 5: coding potential calculator (Kong et al., 2007), Pfam-scan (Bateman et al., 2002), phylogenetic codon substitution frequency (phyloCSF) (Lin, Jungreis & Kellis, 2011), and coding-non-coding-index (Sun et al., 2013) were used for distinguishing mRNAs from lncRNAs.

### Target gene prediction

To discover lncRNA function, the prediction of lncRNA target genes in *cis* and *trans* forms were conducted. We searched for protein-coding genes within 10 and 100 kb upstream and downstream of lncRNAs as the *cis* target gene. Then, the *trans* role of lncRNAs was identified by expression level (Pearson correlation ≥ 0.95 or ≤ −0.95).

### Conserved analysis

To explore transcript conservation, lncRNA and mRNA conservation scores were computed using phastCons (Siepel et al., 2005).

### GO and KEGG enrichment analysis

To explore the functions of the differential lncRNAs, gene ontology (GO) enrichment analysis of differentially expressed lncRNA target genes was implemented by the GOseq R package (Young et al., 2010), in which gene length bias was corrected. GO terms with a corrected *P*-value < 0.05 were considered significantly enriched by differentially expressed genes (DGEs). KEGG pathway analysis was performed to uncover the biological functions of these genes. KOBAS software (Mao et al., 2005) was used to test the statistical enrichment of DGEs or lncRNA target genes in KEGG pathways.

### Real-time quantitative PCR

To validate data accuracy, real-time quantitative PCR (RT-qPCR) was conducted. Total RNA was converted to cDNA using a PrimeScript™ RT Reagent kit (TaKaRa, Dalian, China). RT-qPCR was performed in a LightCycler 480II instrument (Roche, Basel, Switzerland) in reactions containing the SYBR® Green PCR Master Mix (TaKaRa, Dalian, China). The thermal cycler program included an initial denaturation at 95 °C for 3 min, followed by 40 cycles at 95 °C for 15 s; 57 °C for 15 s; and 72 °C for 20 s. All amplifications were followed by dissociation curve analysis of the amplified products. Gene expression was quantified relative to β-actin expression using the 2^−ΔΔCt^ method (Livak & Schmittgen, 2001).

## Results

### Characterization of the spleen transcriptome and identification of lncRNAs

A total of 1,530,737,896 raw reads were produced from 15 porcine spleen samples. After discarding low-quality sequences and adaptor sequences, 1,450,292,484 clean reads (accounting for 216.8 G) were achieved. The percentage of clean reads among raw data in each library ranged from 89.27% to 97.71%. Next, approximately 74.91% of clean reads were mapped to the latest reference genome and about 67% were aligned with unique loci. Most reads were matched mRNA, accounting for 55.32% to 60.28%. Nevertheless, 34.05% to 41.9% of the reads were mapped outside of annotated loci (Table S1).

**Figure 2:**
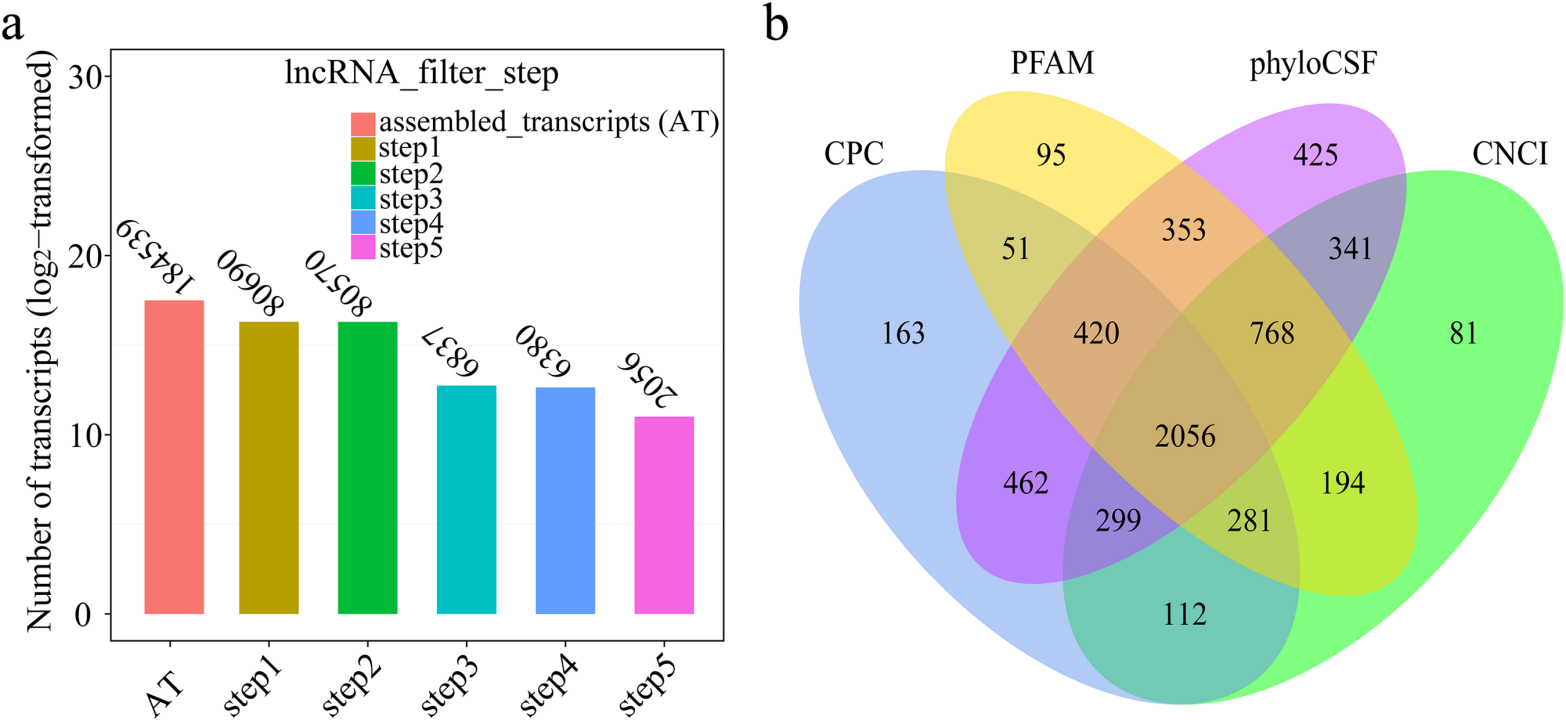
(A) Transcripts number of each step in filtering lncRNAs; (B) Venn diagrams of coding potential analysis by four tools. Those simultaneously shared by four analytical tools were treated as potential lncRNAs.

A total of 184,539 transcripts were initially assembled (Fig. 2A). Eventually, 2,056 novel lncRNAs were identified from spleen samples and subjected to further analysis (Fig. 2B). The 2,056 lncRNAs were classified into 1,811 large intergenic noncoding RNAs and 245 antisense lncRNAs, accounting for 88.1% and 11.9%, respectively. These transcripts corresponded to 1,561 lncRNA genes, an average of 1.6 transcripts per lncRNA locus (Table S2). Interestingly, no intronic lncRNAs were detected.

### Differential expression analysis and RT-qPCR validation

**Figure 3:**
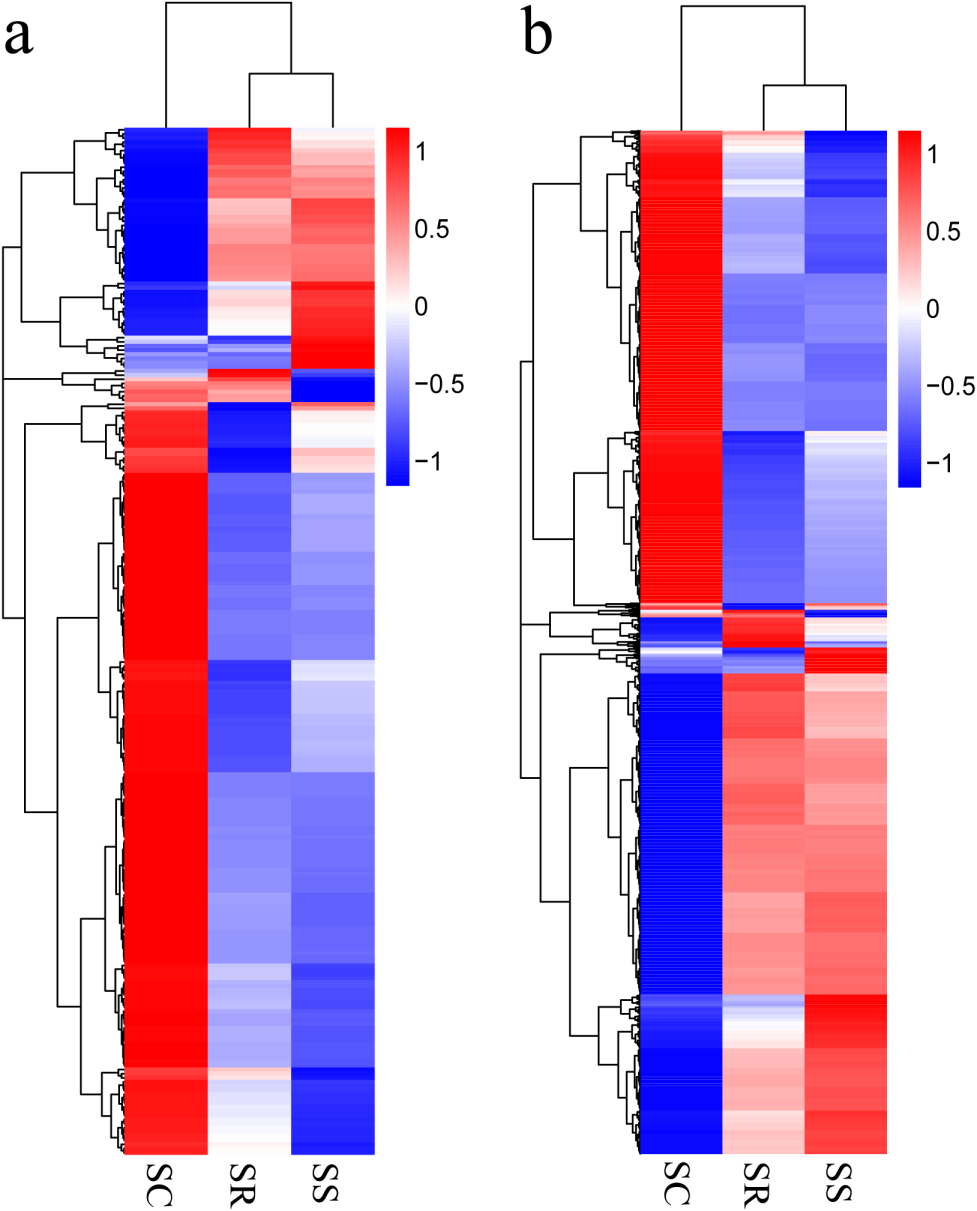
(A) A hierarchical heat map showing the expression value for mRNAs; (B) A hierarchical heat map showing the expression value for lncRNAs.

Transcripts with corrected *P* < 0.05, in pairwise comparison of samples collected from piglets (SS vs. SC; SS vs. SR; SR vs. SC), were assigned as differentially expressed (247 lncRNAs and 2,170 mRNAs). A total of 247 lncRNAs consisted of 123 novel lncRNAs and 124 annotated lncRNAs (Table S3). Furthermore, the differentially expressed mRNAs and lncRNAs in the spleen of the three groups were used in a systematic cluster analysis to analyze similarities. The heat map revealed that SS and SR were clustered together due to their similar expression profiles (Figs. 3A and 3B).

**Figure 4:**
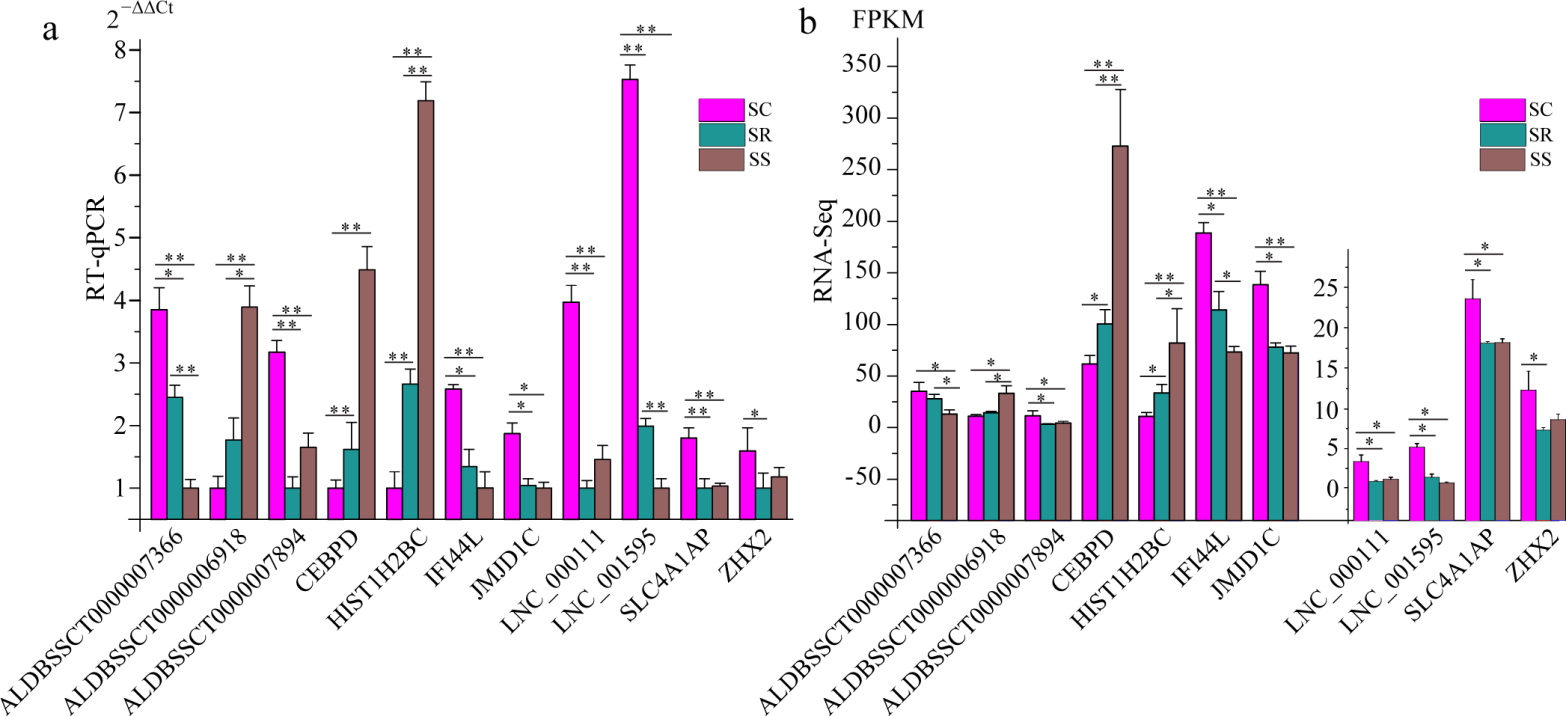
Validation for the RNA-Seq data by RT-qPCR. (A) RT-qPCR results; (B) RNA-Seq results. The results were presented as the mean ± SE (*P < 0.05; **P < 0.01).

**Figure 5:**
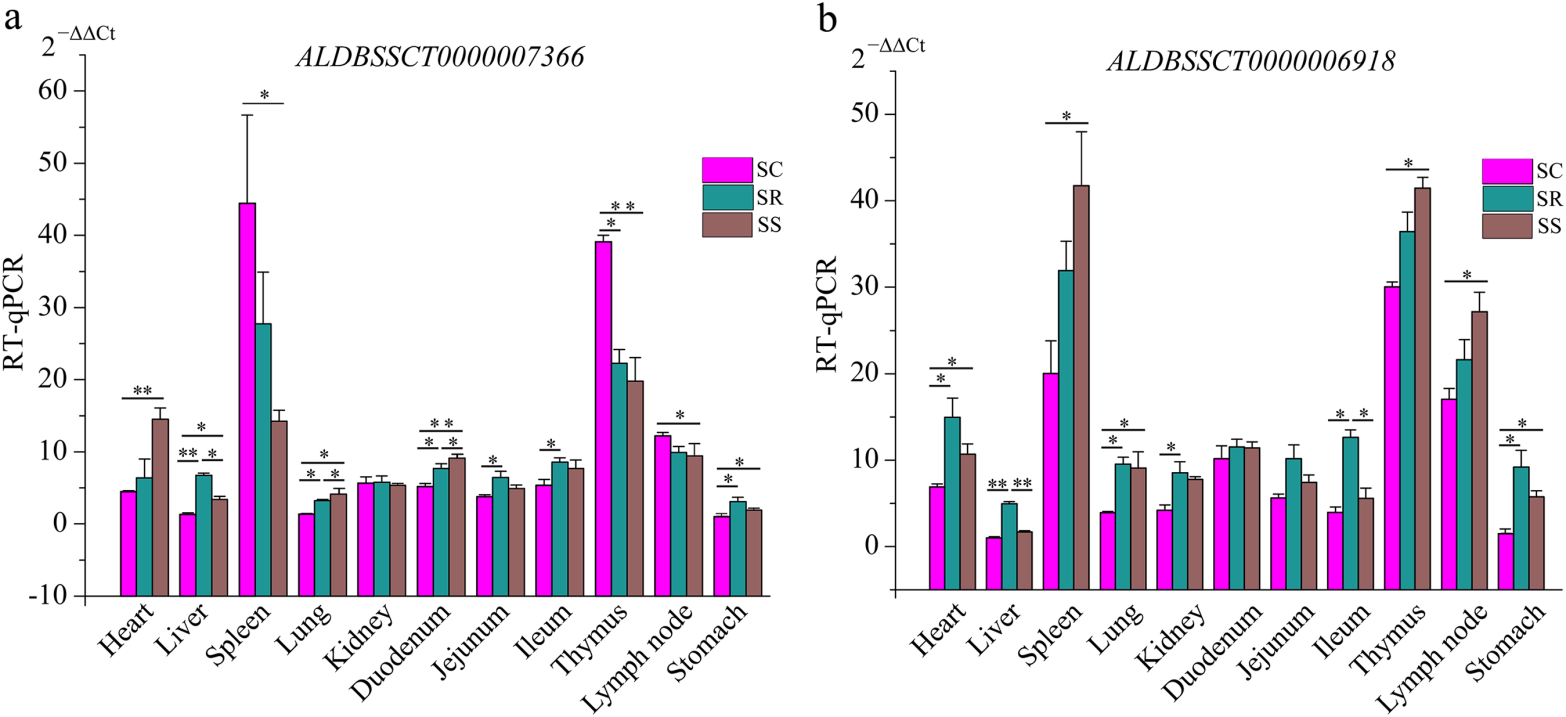
Expression of *ALDBSSCT0000007366* (A) and *ALDBSSCT0000006918* (B) in different tissues using RT-qPCR (**P < 0.01; *P < 0.05).

To validate the RNA-Seq results, six differentially expressed mRNAs and five differentially expressed lncRNAs were selected for RT-qPCR (Table S4). The results showed that expression patterns of selected genes were in agreement with the RNA-Seq data, suggesting that our transcript identifications and abundance estimations were highly reliable (Fig. 4). Compared with protein-coding genes, tissue specificity is an obvious feature of lncRNAs; therefore, we detected the expression of *ALDBSSCT0000007366* and *ALDBSSCT0000006918* in 10 tissues of SS, SR, and SC (Fig. 5). Notably, *ALDBSSCT0000007366* tended to have higher expression levels in spleen than in other tissues. *ALDBSSCT0000006918* was highly expressed in lymph nodes, thymus, and spleen tissue.

### mRNAs and lncRNAs features

**Figure 6:**
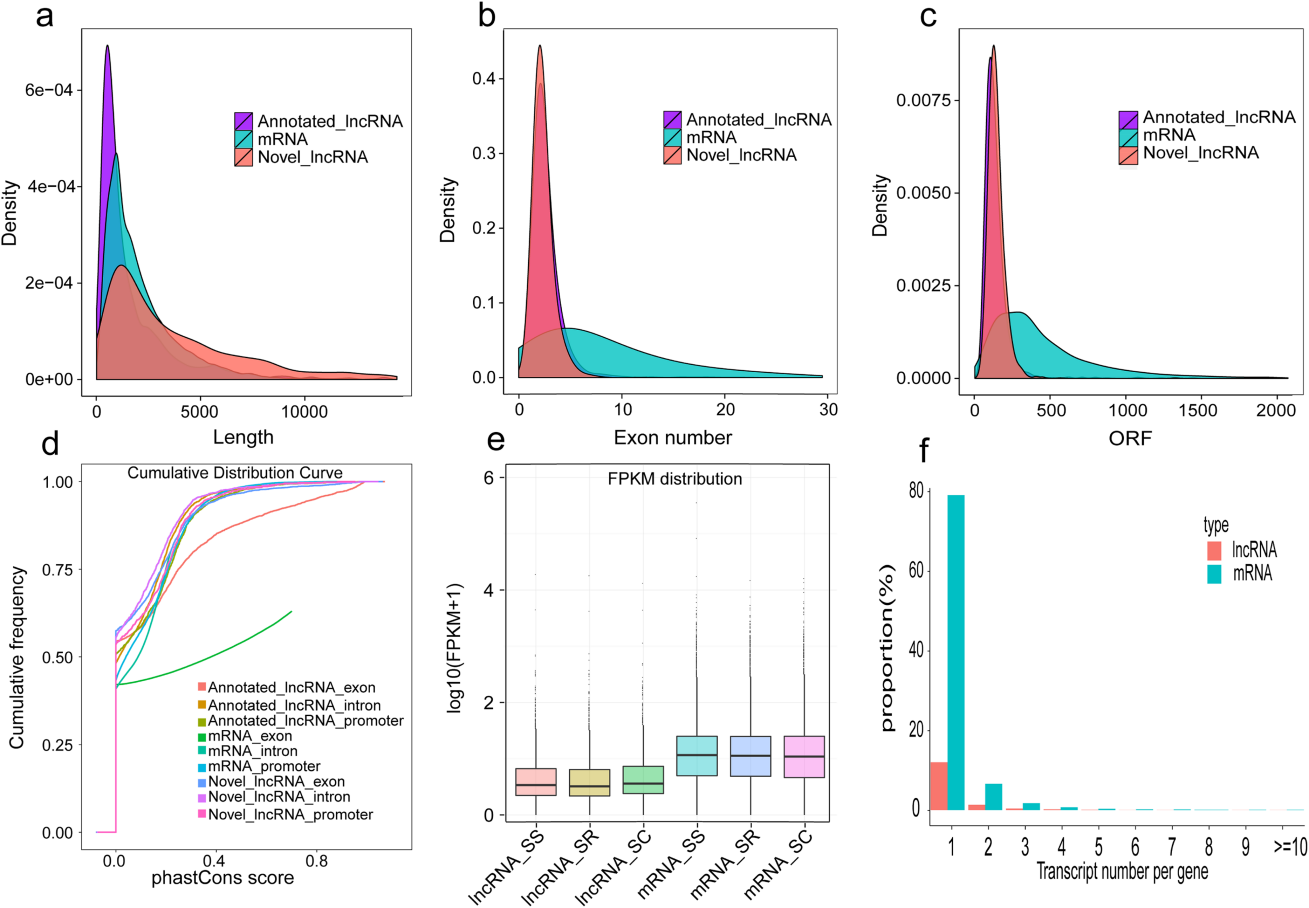
Comparison of genomic features in lncRNAs and mRNAs. (A) Length distribution of annotated lncRNAs, mRNAs and novel lncRNAs in spleen; (B) Exon number distribution of annotated lncRNAs, mRNAs and novel lncRNAs; (C) ORF length distribution of annotated lncRNAs, mRNAs and novel lncRNAs;. (D) Conservation of annotated lncRNAs, mRNAs and novel lncRNAs; (E) Expression level of the mRNAs and lncRNAs among three groups; (F) Proportional distribution of alternative splicing of the mRNAs and lncRNAs.

In our study, a total of 2,144 annotated lncRNAs, 2,056 novel lncRNAs, and 10,081 mRNAs were identified in pig spleen samples. We analyzed the gene structure, expression, and sequence conservation of the lncRNAs and mRNAs to explore differences between annotated lncRNAs, mRNAs, and novel lncRNAs. Our results indicated that (1) there was a distinct difference in the distribution of transcript length between the lncRNAs and mRNAs (Fig. 6A); (2) most of the novel lncRNAs and annotated lncRNAs contained two or three exons despite mRNAs containing more exons (Fig. 6B); (3), most novel lncRNAs and annotated lncRNAs tended to be shorter in ORF (Open Reading Frame) length than most mRNAs (Fig. 6C); (4) most mRNAs were slightly more conserved than lncRNAs. The mRNA exons were more conserved (Fig. 6D); and when compared with mRNAs, the novel lncRNAs and annotated lncRNAs had lower expression levels (Fig. 6E); furthermore, mRNAs produced more alternatively spliced transcripts, in contrast to lncRNAs (Fig. 6F).

### Function enrichment analysis of the *cis* and *trans* role of dysregulated lncRNAs

To explore function of the lncRNAs, the potential targets of lncRNAs in *cis* and *trans* forms were predicted in this study.

The results showed that 3,400 lncRNAs corresponded to 2,821 protein-coding genes within a range of 100 kb, and 1,781 lncRNAs represented 1,867 protein-coding genes within a range of 10 kb (Table S5). GO analysis of *cis* lncRNA targets showed that SR and SS shared some terms such as antigen processing and presentation, the MHC protein complex, and peptide transporter activity (Table S6-1 and S6-2, corrected *P* < 0.05). The significant term was the nucleus-vacuole junction in SR vs. SS (Table S6-3, corrected *P* < 0.05). KEGG pathway analysis of *cis* target genes of lncRNAs revealed that SR and SS shared several pathways such as ABC transporters, antigen processing and presentation, and primary immunodeficiency (Tables S6-4 and S6-5, corrected *P* < 0.05). Interestingly, the significant pathway here was regulation of autophagy in SR vs. SS (Table S6-6, corrected *P* < 0.05).

Subsequently, the *trans* role of lncRNAs in protein-coding genes was investigated. A total of 17,620 interaction relationships were discovered in *trans* between 769 lncRNAs and 3,473 protein-coding genes in the pig genome (Table S7). GO analysis of *trans* lncRNA targets demonstrated that there were 188 and 194 GO terms in SS vs. SC and SR vs. SC, respectively. The top 20 terms were enriched in cellular components and biological processes, such as gene expression, RNA metabolic processes, regulation of macromolecule metabolic processes, and nucleic acid binding in SS vs. SC. The top seven terms were all enriched in cellular components, which participated in nuclei, nucleoplasm, organelle lumina, and so on in SR vs. SC (Tables S8-1 and S8-2, corrected *P* < 0.05). There were no significant terms in SR vs. SS (Table S8-3, corrected *P* < 0.05). However, there were 424 significant GO terms in SR vs. SS, including in defense response to bacterium, regulation of cell adhesion, regulation of humoral immune response, NK T cell activation, and regulation of defense response to viruses (Table S8-3, over-represented *P* < 0.05). KEGG pathway analysis of *trans*-target genes of lncRNAs demonstrated that SR and SS shared some pathways related to immune system function, such as the TNF signaling pathway, NF-kappa B signaling pathway, B cell receptor signaling pathway, MAPK signaling pathway, Alzheimer’s disease, and Huntington’s disease (Tables S8-4 and S8-5, corrected *P* < 0.05). Interestingly, the special pathways were inflammatory bowel disease (IBD), nitrogen metabolism, and vascular smooth muscle contraction in SR vs. SS (Table S8-6, corrected *P* < 0.05).

### Understanding of susceptibility or resistance to *C. perfringens* type C infection

**Figure 7:**
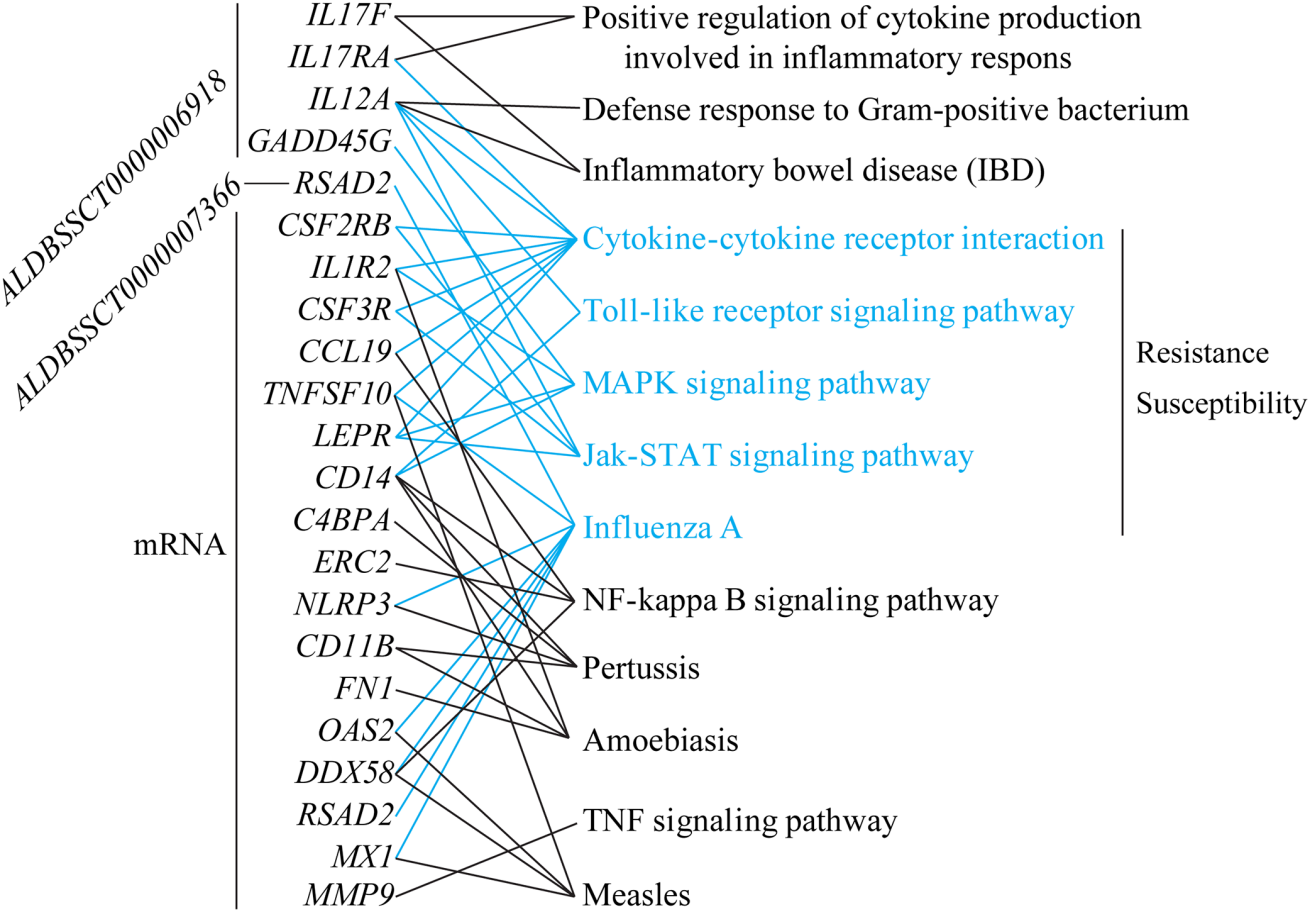
lncRNAs and mRNA associated with susceptibility or resistance to *C. perfringens* type C infection.

To explore differences in resistance and susceptibility to *C. perfringens* type C infection, we analyzed differentially expressed mRNAs and lncRNAs in SR vs. SS according to three criteria. Firstly, lncRNAs were filtered if they had no target genes in *trans* and *cis*. Secondly, target genes of lncRNAs and differentially expressed mRNAs were associated with infectious or immune disease, inflammation, or immune activation (searching for information in Web of Science and GeneCards, Table S9). Lastly, these target genes and DGEs participated in immune/inflammation KEGG pathways. All in all, a total of two lncRNAs, five target genes, and 17 differentially expressed mRNAs were screened. *ALDBSSCT0000006918* targets the four immune-associated genes, *IL17RA* (Interleukin 17 Receptor A), *IL12A* (interleukin 12A), *IL17F* (Interleukin 17F), and *GADD45G* (Growth Arrest and DNA Damage Inducible Gamma); and *ALDBSSCT0000006918* targets the *RSAD2* (Radical S-Adenosyl Methionine Domain Containing 2) gene. In addition, these targets of two lncRNAs and 17 differentially expressed mRNAs were commonly enriched in five important immune-related KEGG pathways, including Toll-like receptor signaling pathway and cytokine–cytokine receptor interaction (Fig. 7). Two lncRNAs could regulate the expression levels of these targets in these KEGG pathways to be involved in *C. perfringens* type C infection. For example, *ALDBSSCT0000006918* regulates the *IL12A* gene in five pathways (such as IBD and Toll-like receptor signaling pathway). These results suggest that these two lncRNAs, via their target genes in immune-related pathways, contributed to susceptibility or resistance to *C. perfringens* type C infection.

**Figure 8:**
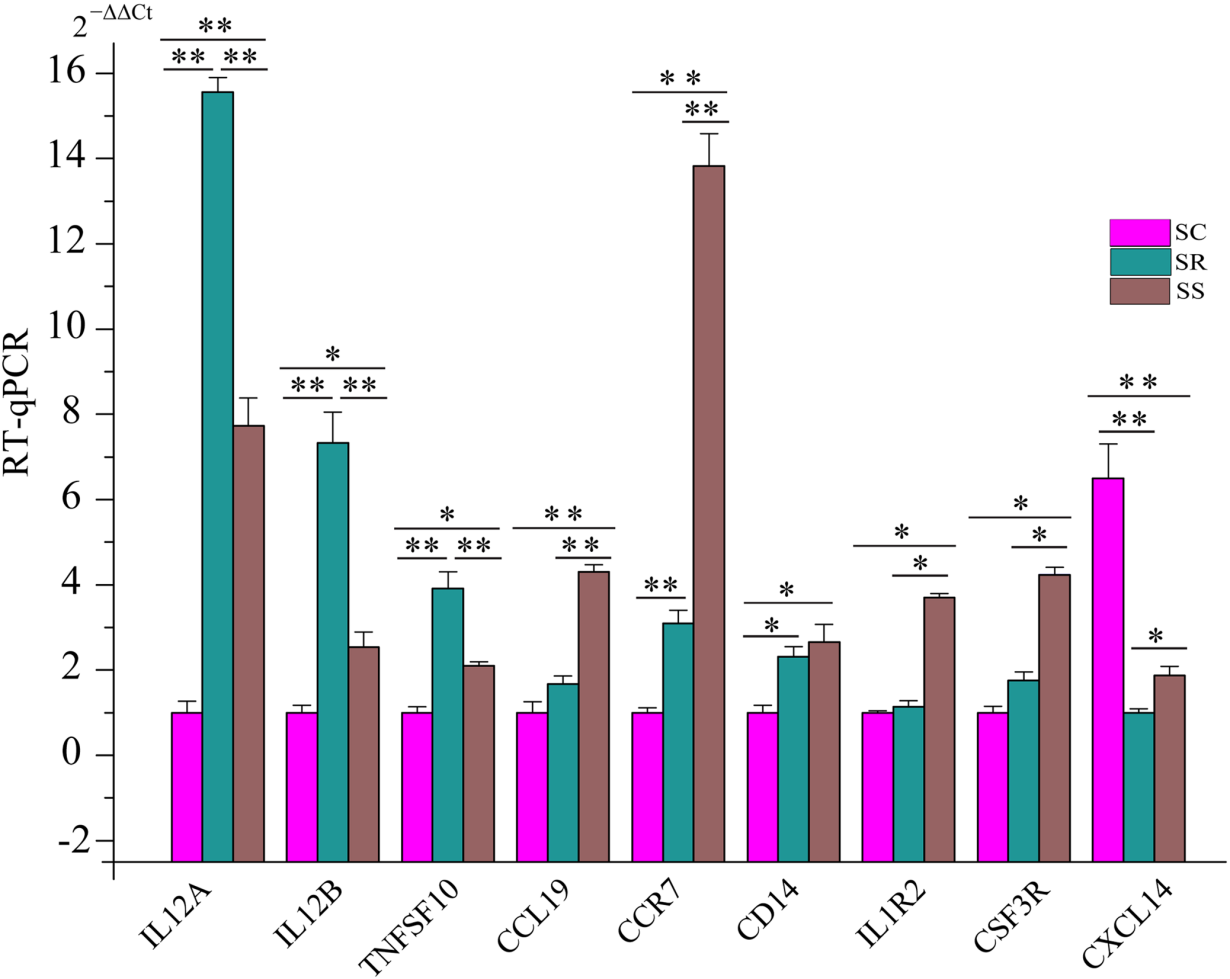
The expression levels of key genes in five pathways associated with susceptibility and resistance to *C. perfringens* type C infection.

To further certify these immune response/inflammation-related pathways, we detected the expression of some key genes in five pathways using RT-qPCR. These genes included *IL12A*, *IL12B* (interleukin 12B), *TNFSF10* (TNF Superfamily Member 10), *CCL19* (C–C Motif Chemokine Ligand 19), *CCR7* (C–C Motif Chemokine Receptor 7), *CD14* (CD14 Molecule), *IL1R2* (Interleukin 1 Receptor Type 2), *CSF3R* (Colony Stimulating Factor 3 Receptor), and *CXCL14* (C-X-C Motif Chemokine Ligand 14). Compared with uninfected piglets in SC, the expression of *CXCL14* was down-regulated. However, expression levels of eight genes increased in SS and SR. Additionally, we also observed that the expression levels of *IL12A*, *IL12B*, and *TNFSF10* were higher in SR than that in SS, and the expression levels of *CCL19*, *CCR7*, *CD14*, *IL1R2*, and *CSF3R* were lower in SR than those in SS, respectively (Fig. 8). These results suggested that these dysregulated genes played important roles in piglet immune systems to combat *C. perfringens* type C after infection.

## Discussion

*Clostridium perfringens* type C is well known as one of the major causative bacteria leading to necrotic enterotoxemia with herd morbidity rates of 30% to 50% in piglets and has a significant impact on the global pig industry (Fisher et al., 2006; Schaefer, Zimmermann & Posthaus, 2013). However, there are no reports about the biological function and significance of mRNAs and lncRNAs in the spleens of piglets with *C. perfringens* type C infection. The current study addressed this gap in scientific knowledge.

Draft spleen sequences were generated from 15 piglets with approximately 466.85, 468.93, and 514.5 million clean reads for SS, SR, and SS, respectively, in which at least 73.49% of the reads aligned to the pig genome (Table S1). This was lower than the percentage obtained from the liver (82.15–84.56%), muscle (77.28–77.86%), fat (77.25–81.59%) (Yu et al., 2017), and thyroid gland (81.50–84.37%) (Shen et al., 2016) transcriptomes; however, this was higher than in fetal porcine skeletal muscle (73.1%) (Zhao et al., 2015). These results may have been caused by different tissues and yield of clean reads. A large proportion of clean reads (34.05–41.9%) were mapped to unannotated loci (Table S1); this suggests that pig genome annotation is not perfect and should be improved. We identified a total of 2,056 novel lncRNAs and annotated 2,144 lncRNAs. The number of lncRNAs in this experiment was much lower than that of porcine lncRNAs (12,103) in the ALDB (Li et al., 2015), and human lnRNAs (146,742) in LNCipedia 4.1 (Volders et al., 2015). It is probable that lncRNAs are expressed in a tissue-specific pattern (Washietl, Kellis & Garber, 2014). Thus, the number of lncRNAs in the spleen is small.

The characteristics of our newly identified lncRNAs in pig spleen tissue were similar to those of other studies (Derrien et al., 2012; Pauli et al., 2012; Wang et al., 2016). They were shorter, had a lower exon number, lower expression, shorter ORF, and were less conserved than those of protein-coding transcripts (Figs. 6A–6E). This similarity of lncRNAs could be related to the important functions of regulation. For example, novel lncRNAs in spleen (4,728 bp on average) were longer than in fetal porcine skeletal muscle (1,043 bp on average), pig endometrium (1,454 bp on average), zebrafish embryo (1,113 bp on average), and pig thyroid gland (2,337 bp on average), and contained fewer exons (two exons on average) than porcine skeletal muscle (2.5 exons on average), zebrafish embryo (2.8 exons on average), pig thyroid gland (2.3 exons on average), and pig endometrium (2.4 exons on average) (Pauli et al., 2012; Shen et al., 2016; Wang et al., 2016; Zhao et al., 2015). Previous studies indicate that a feature of lncRNAs is obvious tissue-specificity compared with mRNAs (Mercer, Dinger & Mattick, 2009; Ponting, Oliver & Reik, 2009). Hence, we selected *ALDBSSCT0000006918* and *ALDBSSCT0000007366* to detect the expression in different tissues and obtained a common conclusion. RNA expression profiling across pig tissues showed that the transcripts *ALDBSSCT0000006918* and *ALDBSSCT0000007366* were expressed in 10 tissues, but they had a higher expression level in immune organs, such as the spleen and lymph nodes (Fig. 5). Expression patterns showed that *ALDBSSCT0000006918* and *ALDBSSCT0000007366* may have important roles in immune function.

To determine why some piglets are resistant to *C. perfringens* type C, and others are not, we analyzed DEG mRNAs and lncRNAs in SR vs. SS. Based on three strict criteria, we identified that two lncRNAs and 17 genes were associated with susceptibility and resistance to *C. perfringens* type C infection. In the filtered gene pairs, the differentially expressed *ALDBSSCT0000006918* and *ALDBSSCT0000007366* regulated some immune-related genes: *IL12A*, *IL17F*, *GADD45G*, *IL17RA*, and *RSAD2* (Fig. 7). Compared to SC, *ALDBSSCT0000006918* expression level was higher in the spleen samples of SS and SR. In addition, *ALDBSSCT0000007366* expression level was lower in the spleen samples of SS and SR compared with SC (Fig. 4). These results showed that they had regulated function in the spleens of piglets infected with *C. perfringens* type C. Interestingly, target genes of two lncRNAs were involved in IBD and defense response to Gram-positive bacterium. In addition, four pathways were shared (Toll-like receptor signaling pathway, cytokine–cytokine receptor interaction, MAPK signaling pathway, and Jak-STAT signaling pathway) related to *C. perfringens* type C infection; these were the same as the conclusions of spleens in *C. perfringens* type A infected chicken (Truong, Hong & Lillehoj, 2015) and in *E. coli* infected broilers (Sandford et al., 2011). To thoroughly understand whether these pathways are involved in *C. perfringens* type C infection, we evaluated the expression of several key genes of these KEGG pathways. *IL12A* participated in IBD, cytokine–cytokine receptor interaction, Toll-like receptor signaling pathway, and Jak-STAT signaling pathway. *IL12A* (p35) can bind to *IL12B* (p40) to form *IL12* (p35/p40 complex), which plays a key role in enhancing cellular immune function and anti-pathogenic microorganisms (Jung et al., 2018; Teng et al., 2015). *IL12A* and *IL12B* were more significantly expressed in SR than that in SS groups after *C. perfringens* type C infection. These results showed that high expression of *IL12A* and *IL12B* in these pathways is beneficial to resisting *C. perfringens* type C. *TNFSF10* (*TRAIL*) can be expressed in different immune cells (such as NK cell, T cell, and macrophage cell) and plays an important role in immune modulation, pathogen resistance, and tumor surveillance (Ikeda et al., 2010; Jin & El-Deiry, 2006). *TNFSF10* is an important part in the cytokine–cytokine receptor interaction pathway and influenza A pathway. Studies have already found that higher expression of *TNFSF10* (TRAIL) can suppress HIV infection (Nie et al., 2018). In our study, *TNFSF10* expression was up-regulated in SR and SS compared with SC, being particularly higher in SR. This indicated that up-regulated *TNFSF10* may inhibit *C. perfringens* type C invasion and reproduction*. CCL19* is one of several cytokine genes and is related to immune processes, including normal lymphocyte recirculation, T cell and B cell migration by specifically binding to *CCR7* (Aucott et al., 2016)*. CCL19* and *CCR7* were differentially expressed in SR and SS, being particularly higher in SS. This showed that lower expression of *CCL19* and *CCR7* could suppress this bacterium. *CXCL14* is a chemokine with a wide range of biological functions, mainly playing roles in immune cell migration and antimicrobial immunity (Lu et al., 2016; Sidahmed et al., 2014). A previous study found that line-6 chickens (more susceptible to *C. perfringens* type A) had a significantly down-regulated *CXCL14* level over that of line-7 chickens (more resistant to *C. perfringens* type A) (Kim et al., 2014). Indeed, we found that the expression of *CXCL14* was significantly decreased in SS over SR. All in all, these results indicated that *ALDBSSCT0000006918*, *ALDBSSCT0000007366*, their targets, and these DGEs were most probably involved in resisting *C. perfringens* type C.

Our study has initially indicated which mRNAs and lncRNAs are likely to assist in resisting *C. perfringens* type C. However, future studies are needed to explore the concrete role of the two lncRNAs in cell lines exposed to *C. perfringens* type C or its products.

## Conclusions

We first addressed the expression profile of lncRNAs in the spleens of three groups of piglets (SR, SS, and SC) infected with *C. perfringens* type C. We found that two lncRNAs (*ALDBSSCT0000006918* and *ALDBSSCT0000007366*) contributed to susceptibility and resistance to *C. perfringens* type C infection.

## Supplemental Information

10.7717/peerj.5997/supp-1 Supplemental Information 1 Table S1. The information of the RNA-Seq data.

10.7717/peerj.5997/supp-2 Supplemental Information 2 Table S2. Analysis of the subtypes of novel lncRNAs in transcript length, exon number, ORF and FPKM.

10.7717/peerj.5997/supp-3 Supplemental Information 3 Table S3. Differential expression genes in three comparison groups.

10.7717/peerj.5997/supp-4 Supplemental Information 4 Table S4. Primer sequence for the selected genes.

10.7717/peerj.5997/supp-5 Supplemental Information 5 Table S5. The protein-coding genes within 10k and 100k upstream and downstream of lncRNAs.

10.7717/peerj.5997/supp-6 Supplemental Information 6 Table S6. Functional enrichment analysis of the protein-coding genes of lncRNAs in *cis*.

10.7717/peerj.5997/supp-7 Supplemental Information 7 Table S7. Pearson correlations between protein-coding genes and lncRNAs.

10.7717/peerj.5997/supp-8 Supplemental Information 8 Table S8. Functional enrichment analysis of the protein-coding genes of lncRNAs in *trans*.

10.7717/peerj.5997/supp-9 Supplemental Information 9 Table S9. The understanding of susceptibility or resistance to C. perfringens type C infection.

## Additional Information and Declarations

### Field Study Permissions

The following information was supplied relating to field study approvals (i.e., approving body and any reference numbers): Sample collection was approved by the ethics committee of Gansu Agricultural University.

### Data Availability

The following information was supplied regarding data availability: The sequencing data were submitted to the Genome Expression Omnibus (accession number GSE105797) in NCBI.
